# T Cell Metabolism in Cancer Immunotherapy

**DOI:** 10.20900/immunometab20200020

**Published:** 2020-06-10

**Authors:** Halil-Ibrahim Aksoylar, Natalia M. Tijaro-Ovalle, Vassiliki A. Boussiotis, Nikolaos Patsoukis

**Affiliations:** 1 Division of Hematology-Oncology, Beth Israel Deaconess Medical Center, Harvard Medical School, Boston, MA, 02215, USA; 2 Department of Medicine, Beth Israel Deaconess Medical Center, Harvard Medical School, Boston, MA 02215, USA

**Keywords:** T cell differentiation, immunometabolism, cancer immunotherapy, T cell memory, glycolysis, mitochondria, ROS, adoptive cell therapy

## Abstract

Immune checkpoint therapies aiming to enhance T cell responses have revolutionized cancer immunotherapy. However, although a small fraction of patients develops durable anti-tumor responses, the majority of patients display only transient responses, underlying the need for finding auxiliary approaches. Tumor microenvironment poses a major metabolic barrier to efficient anti-tumor T cell activity. As it is now well accepted that metabolism regulates T cell fate and function, harnessing metabolism may be a new strategy to potentiate T cell-based immunotherapies.

## INTRODUCTION

Metabolic reactions that occur in living cells result in the generation of adenosine triphosphate (ATP), the central molecule that fuels most energetic processes. Cells use carbohydrates as substrates for energy by engaging glycolysis, the anaerobic pathway that takes place in the cytoplasm and converts glucose to pyruvate. Pyruvate is converted to lactate by lactate dehydrogenase with concomitant interconversion of NAD^+^ and NADH. Pyruvate can also be converted to acetyl-CoA and enter the tricarboxylic cycle (TCA cycle), known as the Krebs cycle, which occurs in the mitochondria and requires the presence of oxygen. The disposal of electrons released by both glycolysis and TCA cycle generates ATP in a series of reactions known as oxidative phosphorylation (OXPHOS) in the electron transport chain (ETC). Although glycolysis and lactate production (also known as lactate fermentation) is an anaerobic process, it can occur also in presence of ample amounts of oxygen. This form of aerobic glycolysis is known as the Warburg effect [1].

An often-neglected part of metabolism tightly connected with the main metabolic pathways is metabolism of reactive oxygen species (ROS). ROS are formed mainly in mitochondria when electrons escape the ETC and combine with oxygen to form unstable and highly reactive forms of oxygen, which are involved in the regulation of biological processes. For example, ROS-mediated reversible oxidation of protein thiols has been implicated in the regulation of phosphatases, kinases, transcription factors, epigenetic regulators and antioxidant enzymes [2–10]. A central regulator of cellular resistance to oxidants is transcription factor Nrf2, which is kept suppressed by Keap1-dependent ubiquitination and proteasomal degradation. Under conditions of oxidative stress, Keap1 oxidation results to Nrf2 release and translocation to the nucleus [11], where it controls an array of antioxidant response element (ARE)-dependent genes to provide a permissive setting for exposure to an oxidative environment [11–14]. Disturbance of the normal redox state can lead to damaged proteins, lipids, and DNA [15], however, moderate ROS levels can also act as important messengers in redox signaling.

T cells are specific effectors of our immune system, which, besides pathogens, they continuously survey and eliminate tumor cells [16]. However, major obstacles such as the expression on T cell surface of inhibitory receptors (known as checkpoint inhibitory molecules) as well as the metabolically hostile tumor microenvironment prevent anti-tumor function. Antibody-mediated blockade of T cell inhibitory receptors (termed immune checkpoint therapy; ICT), has shown promise to enhance T cell responses against cancer. However, ICT alone has been largely unsuccessful [17–20] as only a fraction of patients develops durable antitumor responses, underlying the need for finding new combinatorial strategies to improve ICT outcomes. Here we will discuss research findings on how metabolic interventions may synergize with ICT to improve T cell-based tumor immunotherapies.

## METABOLIC ADAPTATIONS DURING DIFFERENTIATION OF NAïVE INTO EFFECTOR T CELLS

**Figure 1.**
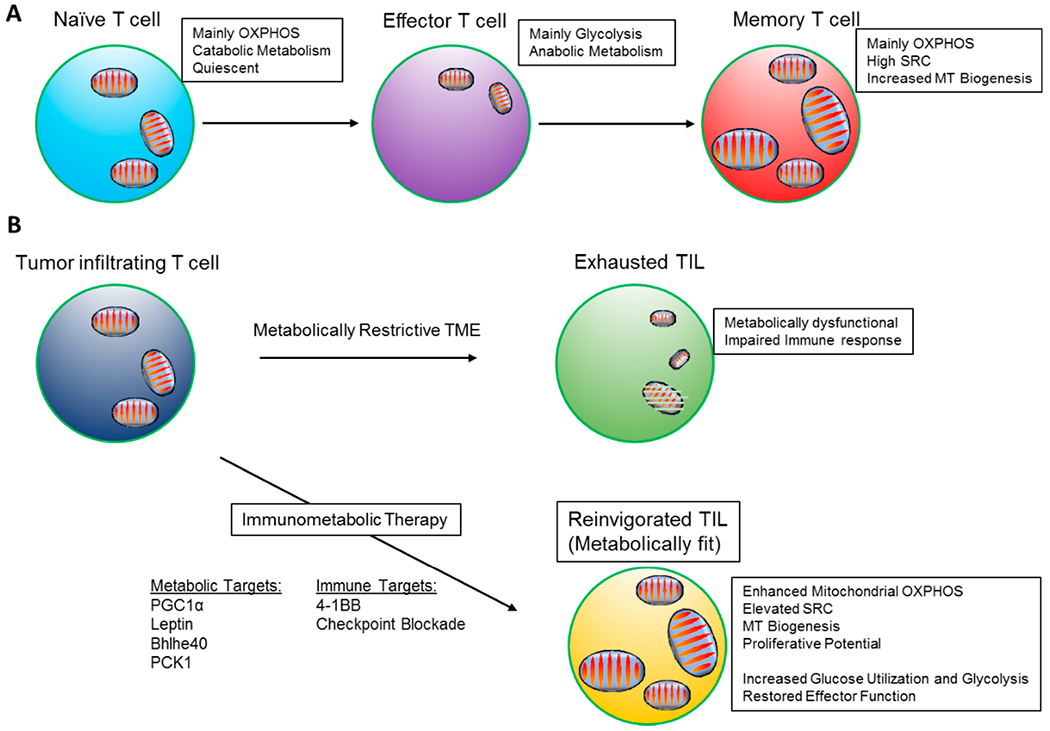
Mitochondrial metabolism supports T cell responses. **(A)** Metabolic states of naïve, effector and memory T cells. **(B)** Dysfunctional metabolism in exhausted TILs with loss of mitochondrial fitness. Potential molecular targets to be activated or overexpressed to reinvigorate mitochondrial metabolism and to enhance anti-tumor function.

Circulating in the lymphoid tissues, naïve T cells utilize a slow rate of metabolic activity to meet their energy demands for survival and a slow rate of homeostatic proliferation. Naïve T cells are quiescent, and their low metabolic needs are sustained by mitochondrial metabolism. They generate ATP mainly through oxidation of pyruvate in the TCA cycle, OXPHOS and fatty acid oxidation (FAO) (Figure 1A) [21–23]. In this resting state, they display lower glucose and fatty acid uptake, and smaller mitochondrial mass compared with resting memory T cells [24].

After encountering antigens that are recognized by the T cell receptor (TCR) and simultaneously activate costimulatory signals such as CD28, T cells undergo extensive proliferation, growth and differentiation into T effector cells [25]. The transition to an effector state is characterized by a shift towards a predominantly glycolytic state that allows T effector cells to fulfill their bioenergetic needs to support generation of biomass and energy required for rapid proliferation (Figure 1A) [26]. Even though activated lymphocytes are able to increase OXPHOS acutely [27–29], engaging glycolysis yields ATP at a sufficient rate and provides key intermediates for the pentose phosphate pathway (PPP), which sustains biosynthesis and generates NADPH, an essential reducing molecule [30]. These metabolic reprogramming events are transcriptionally regulated upon T cell receptor signaling that promotes the expression of amino acid and glucose transporters [30,31]. By using glucose to fulfill their bioenergetic demands, activated T cells spare other nutrients such as amino acids and fatty acids as building blocks for their growth, division and clonal expansion. Paradoxically, although effector T cells have increased fatty acid uptake, they rely on the energy-consuming process of *de novo* fatty acid synthesis to support lipid biosynthesis for building new membranes and generating signaling molecules [32,33]. Notably, it was demonstrated that Th17 but not Treg differentiation depends on acetyl-CoA carboxylase 1 (ACC1), a key enzyme that mediates de novo fatty acid synthesis and targeting ACC1 was proposed as a new strategy for metabolic immune modulation against autoimmune and inflammatory diseases that are mediated by Th17 cells [34].

The PI3K-Akt pathway regulates glycolysis and protein metabolism in activated T cells by phosphorylating the mammalian target of rapamycin (mTOR) [35,36]. When mTOR is inhibited, glycolysis is suppressed and FAO is enhanced, resulting in impaired effector differentiation and enhanced memory phenotype [37]. This has also been observed in murine CD8^+^ T cells, where glucose starvation limits IFN-γ gene expression, and also impairs the transition to T effector phenotype [38]. Therefore, activated T cells have to adapt swiftly to antigen stimulation and upregulate the expression of glucose receptor Glut1, among other nutrient receptors, in order to support anabolic growth [39–41]. During T cell activation and differentiation, expression of glycolysis-related genes and enzymes is also enhanced [30,42,43]. While effector T cells express high levels of glucose transporter Glut1, regulatory T cells (Treg) which have a quiescent phenotype, depend on high lipid oxidation rates promoted by AMP-activated kinase (AMPK) activity, which opposes mTOR-dependent cell growth pathways including de novo fatty acid synthesis [39,44].

Carbohydrates are not the only key nutrients required for T cell activation and effector differentiation. Amino acid metabolism has an indispensable role in the T cell activation process, particularly during antigen encounter and clonal expansion [45,46]. Glutamine is used as a fuel for mitochondrial oxidation, which promotes T effector generation and fitness [47,48]. Glutaminolysis allows ATP production in rapidly proliferating cells and supports their development and functionality, by increasing IL-2 receptor expression and cytokine production [31,49]. Deleting glutamine/leucine transporter Slc7a5 in T cells impaired metabolic reprogramming and interfered with T helper differentiation and clonal expansion [31]. Extracellular alanine deprivation during the early activation phase also led to functional impairment in T cells [50].

## MITOCHONDRIAL METABOLISM REGULATES MEMORY T CELL RESPONSES

Studies investigating the metabolism of memory T cells have demonstrated that spare respiratory capacity (SRC), the extra mitochondrial capacity available in the cell to produce energy under conditions of stress, is critical for memory CD8^+^ T cell differentiation (Figure 1A). Distinct from effector T cells, IL-15-induced memory CD8^+^ T cells display enhanced oxidative metabolism largely due to increased mitochondrial biogenesis and increased expression of carnitine palmitoyl transferase alpha (CPT1α), a rate-limiting metabolic enzyme for mitochondrial FAO (Figure 1A) [51]. Notably, memory T cells utilize FAO to support their development and long-term survival without depending on extracellular fatty acids. Instead, memory CD8^+^ T cells take up extracellular glucose and glycerol to synthesize fatty acids and triglycerides in order to support FAO. Then, the lipolytic enzyme lysosomal acid lipase (LAL) mobilizes stored fatty acids for oxidation and memory T cell development [33,52]. Having an increased mitochondrial mass and enhanced SRC, allows memory T cells to rapidly respond to an antigen-mediated rechallenge.

Among naïve, central and effector memory T cell populations, effector memory T cells are the ones predominantly enriched in the tumor microenvironment and although do not proliferate well relative to naive or central memory T cells, they have enhanced effector functions such as cytotoxic potential and effector cytokine production. Importantly, a recent study identified significant differences in the mechanistic dependency of naïve and central memory T cells on fatty acid metabolism compared with effector memory T cells [53]. Specifically, under glucose starvation, naïve and central memory T cells survived by upregulating fatty acid synthesis, FAO and OXPHOS which however compromised IFN-γ expression upon T cell activation. In contrast, effector memory T cells, although maintained FAO, did not upregulate fatty acid synthesis, which allowed sustained production of high levels of IFN-γ. These observations suggest that effector memory T cells adapt to limited dependency on fatty acids in order to maintain functionality under limiting glucose conditions [53].

Although, several studies support the concept that mitochondrial oxidative metabolism promotes memory T cell development and maintenance, other studies have shown that constitutive glycolysis and memory T cell development may co-exist. Using a conditional deletion model of Von Hippel-Lindau (Vhl), a regulator of HIF1α, Phan and colleagues demonstrated that constitutive activation of HIF1α induced constitutive glycolysis in transgenic T cells. Upon viral infection, VHL-deficient T cells were able to generate long term memory T cells without utilizing mitochondrial metabolism and without possessing increased SRC. Indeed, VHL-deficient memory T cells displayed an effector memory phenotype characterized by T-bet expression and low levels of surface CD62L. This study further demonstrated that, SRC is a characteristic feature of central memory T cells, while effector memory T cells that develop without engaging mitochondrial oxidative metabolism can still provide protective immunity [54].

## ROLE OF MITOCHONDRIA IN T CELL EFFECTOR FUNCTION

Although most studies have focused on the role of glycolysis in effector function and mitochondrial OXPHOS as a means to induce memory and Treg cells, it is increasingly appreciated that both these key metabolic pathways are required to orchestrate T cell effector function, and that increased OXPHOS together with increased glycolysis is a signature of T effector cells. A recent study showed that TCR engagement results in reprogramming of the T cell proteome and phosphoproteome, where the significant increase in mitochondrial functions assists T cell exit from quiescence and entry into the cell cycle, through mTORC1-dependent mitochondrial biogenesis [55]. Notably, another study showed that *in vivo* activated CD8^+^ T cells, in contrast to in vitro stimulation, operated at approximately 50% of maximal glycolysis and had decreased lactate production, particularly at the peak of their expansion phase, while displaying increased rates of oxidative metabolism [56]. Strikingly, increased OXPHOS favored differentiation to Th17 phenotype as TCR-dependent induction of the Th17 transcription factor basic leucine zipper transcription factor TF-like (BATF) was partially regulated by mTORC1 activation, which required ATP-linked mitochondrial OXPHOS [57]. This effect might be partially dependent on mitochondria-driven ROS necessary to support TCR signaling for subsequent transition of quiescent naïve T cells into an activated state [58].

## TUMOR IMMUNOTHERAPY AND T CELL METABOLISM

### Effects of Immune Checkpoint Therapy on T Cell Metabolism

Understanding the role of metabolism in T cell differentiation and function might lead to new interventions to fight cancer. The recent development of monoclonal antibodies against PD-1 and CTLA-4, known as immune checkpoint inhibitors, has revolutionized cancer management in the last decade [17,59,60]. PD-1 blockade rescues exhausted T cells that, upon chronic antigen stimulation, lose their effector function [61,62]. Understanding the metabolic impact of immune-therapies has become critical because partially exhausted T cells display a defective metabolic profile, which can be reverted upon PD-1 blockade treatment [63,64]. PD-1 and CTLA-4 receptors decrease glucose uptake, inhibit glycolysis and impair T cell activation [65], whereas only PD-1 engagement promotes FAO and enhances lipolysis [63,65]. In mice, PD-1 blockade reverses glucose restriction in TILs, enhancing CD8^+^ T cells glucose influx and glycolysis via mTOR signaling, which allows IFN-γ production, improving their effector anti-tumor function [66]. Another study identified the glycolytic metabolite phosphoenolpyruvate (PEP) as a repressor of sarco/ER Ca^2+^-ATPase (SERCA) activity resulting in sustained TCR-mediated Ca^2+^-NFAT signaling and effector functions. Enhancing PEP production by overexpressing PEP carboxykinase 1 (PCK1) potentiated T cell anti-tumor activity, restricting tumor growth and prolonging survival of melanoma-bearing mice [67].

### Manipulation of Glycolytic Metabolism to Improve T Cell Function

Excessive and sustained glycolysis results in terminal differentiation and T cell exhaustion [68]. Limiting glycolysis might be a strategy to enhance memory T cell formation. Using an inhibitor of glycolysis, 2-deoxyglucose, while activating CD8^+^ T cells, enhanced not only the generation of memory cells but also improved anti-tumor activity [68]. Moreover, pharmacologic inhibition of Akt enhanced expansion of potent tumor-specific lymphocytes that express memory T cell markers such as elevated SRC and enhanced lipid oxidation metabolism [69]. These observations indicate that impeding the aggressive glycolytic metabolism either by directly inhibiting anabolic metabolism with glucose analogs or by inhibiting signaling pathways downstream of TCR, enables the formation of memory T cells with potent anti-tumor function. Likewise, inhibition of mTORC1 pathway in viral infection models enhanced the quantity and quality of memory CD8^+^ T cells, largely due to limiting the activation of glycolytic machinery [37].

### Improving Mitochondrial Metabolism to Enhance T Cell Function

Recent studies highlight the importance of mitochondrial fitness and oxidative metabolism for enhanced anti-tumor function by tumor infiltrating T cells. A stress responsive transcription factor, basic helix-loop-helix family member e40 (Bhlhe40), has recently been shown to be a promising target to improve mitochondrial fitness in TILs as Bhlhe40 deficiency abrogated the therapeutic effects of anti-PD-L1 blockade (Figure 1B). In addition, TILs deficient for Bhlhe40 had impaired ability to produce metabolites required for acetyl-CoA synthesis, resulting in decreased histone acetylation of functional genes [70]. Pathways regulating the biogenesis of mitochondria have important implications on anti-tumor function of T cells. Scharping et al. have shown that T cells infiltrating tumors show decreased mitochondrial function and mass due to chronic Akt-mediated inhibition of PPAR-gamma coactivator 1 alpha (PGC1α). When mitochondrial biogenesis was improved by enforced PGC1α expression, tumor specific T cells showed enhanced anti-tumor function, suggesting PGC1α as a promising target to promote T cell fitness (Figure 1B) [71]. In human melanoma tumors, PGC1α expression similarly provides increased mitochondrial capacity and resistance to oxidative stress [72]. Thus, it remains to be seen whether using PGC1α activators or inhibitors would have anti-tumor effects or would potentially support tumor growth.

Besides checkpoint blockade, activation of costimulatory receptors induces metabolic reprogramming with implications on T cell fate and function. 4–1BB costimulation can enhance mitochondrial capacity in CD8^+^ T cells by engaging PGC1α-mediated signaling pathways. Combined with PD-1 blockade, 4–1BB agonist enhanced anti-tumor immunity in B16-F10 melanoma model (Figure 1B) [73]. In a different study, metabolic dysfunction of TILs was overcome by elevating leptin levels in the tumor microenvironment. Delivery of an engineered oncolytic vaccinia virus expressing recombinant leptin improved the anti-tumor function and memory response by TILs through enhanced mitochondrial oxidative metabolism (Figure 1B) [74].

### Balancing Metabolism of ROS in Favor of T Cell Function

Although the tumor microenvironment is characterized by elevated ROS levels [75], use of antioxidants has not been an effective strategy to prevent tumor growth. On the contrary, antioxidants could promote melanoma metastasis [76] and accelerate lung cancer progression in mice [77]. In another study, conditions of increased oxidative stress could inhibit melanoma distant metastasis without affecting tumor growth [78]. Although high supplementation of the powerful antioxidant vitamin C could selectively kill KRAS and BRAF mutant colorectal cancer cells directly, this effect was not dependent on antioxidant function but, in contrast, was mediated by the oxidized form of vitamin C (dehydroascorbate) that depleted GSH resulting in increased production of ROS that inhibited GAPDH, a critical glycolytic enzyme necessary for tumor growth [79]. In addition, vitamin C efficiently controlled aberrant T cell activation by stabilizing CD8^+^ iTregs and enhanced their therapeutic potential in controlling murine GvHD and leukemia relapse [80,81], effects that would not be desired in the context of cancer.

**Figure 2.**
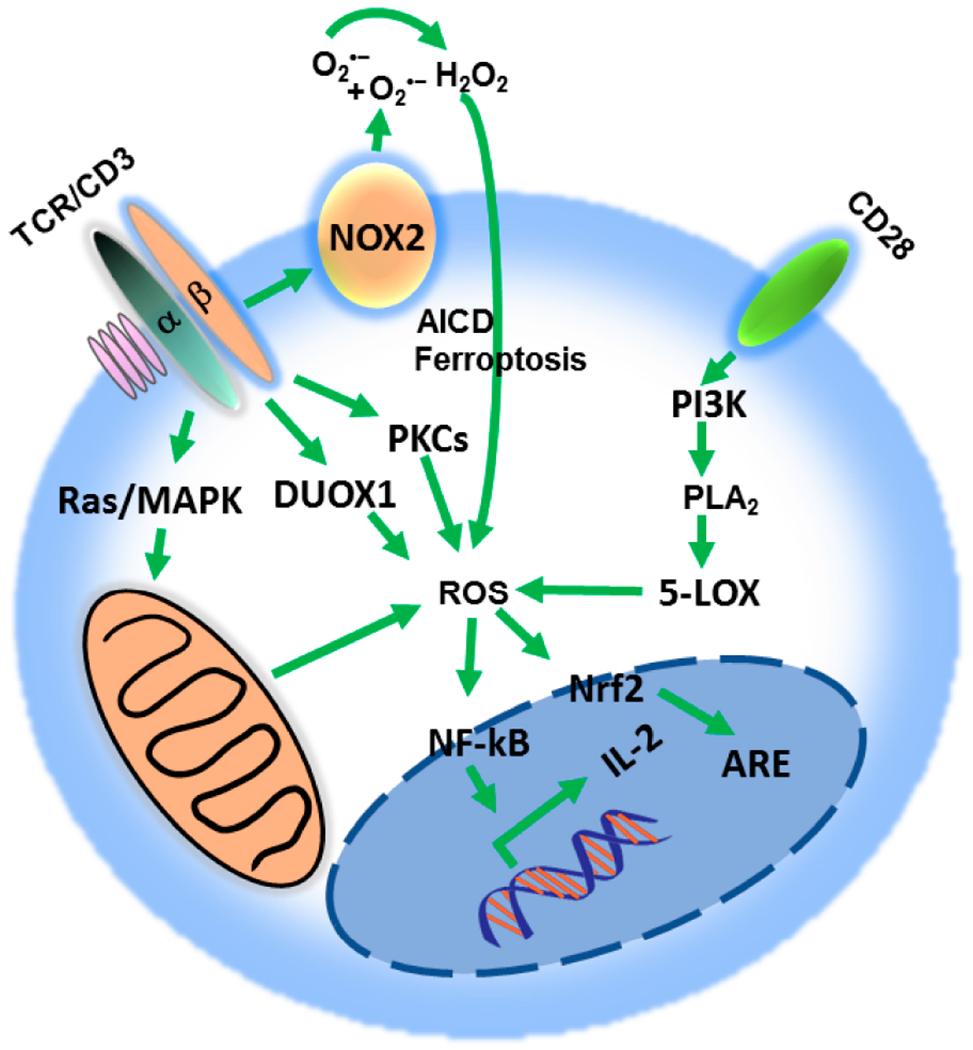
Reactive oxygen species (ROS) impact on T cell activation. T cell receptor (TCR) signaling regulates ROS production by inducing distinct pathways, including phosphorylation of MAPKs cascade, activation of the proximal Duox1 and increase in protein kinase C (PKC)-dependent activation of NADPH oxidase. Co-stimulatory signaling through CD28 activates the PI3K pathway, which generates ROS in the conversion of arachidonic acid intermediates. Moderate levels of superoxide radical (O2^•−^) and hydrogen peroxide (H_2_O_2_) enhance IL-2 transcription, through NFAT nuclear localization, promoting T cell proliferation and activation. ROS induce Nrf2 translocation to the nucleus to regulate antioxidant response element (ARE)-dependent genes. Excessive ROS production results in activation-induced cell death (AICD) or ferroptosis.

Moderate ROS production in T cells is required for cellular and signaling processes leading to T cell activation [82,83]. Complexes I/III of the mitochondrial electron transport chain (ETC) and the enzymes NADPH oxidase (NOX) and 5-lipoxygenase (5-LOX) are the main sources of TCR-induced increase of ROS (Figure 2) [58,82–86]. Consistent with a key role of ROS in T cell responses, T cells deficient for the antioxidant peroxiredoxin have increased proliferation in vitro and in vivo, and enhanced generation of T effector cells [87]. In contrast, antioxidants have been shown to impair T cell responses [88–90]. Thiol redox state (TRS) is regulated by the key antioxidant glutathione (GSH), glutathione disulfide (GSSG), cysteine (CSH) and protein thiols (PSH) [91] and is an essential indicator of the overall cellular redox state and plays an important role in T cell function [89,92].

Upon T cell activation mitochondria translocate proximal to the TCR [93]. From there, mitochondrial ROS (mROS) modulate redox-sensitive kinases and phosphatases to induce activation of NFAT, IL-2 production and proliferation [58,94]. Moderate levels of mitochondrial ROS can promote longevity and response capacity of memory T cells [95]. NOX-derived ROS participate in TCR-signaling at multiple levels. Cytoplasmic NOX member dual oxidase 1 (Duox1) is involved in proximal TCR signaling whereas the membrane-bound NOX2 molecule is activated under conditions of chronic TCR stimulation and is involved in activation-induced cell death (AICD) which requires CD95/CD95L engagement and serves as a controlled cell death mechanism mediating contraction phase of activated T cells (Figure 2) [84,96]. Phospholipase A2 (PLA2) is an enzyme which upon T cell activation provides arachidonic acid for the biosynthesis for various lipids. Cyclooxygenases and lipoxygenases convert arachidonic acid to prostaglandins and leukotrienes, respectively, and these reactions give rise to lipid peroxides and intracellular ROS (Figure 2) [82]. Excessive iron-dependent lipid peroxidation results in a form of cell death termed ferroptosis (Figure 2) [97]. Ferroptosis can be induced in T cells by deficiency in the detoxifying enzyme glutathione peroxidase resulting in membrane accumulation of lipid peroxides and cell death [98]. However, efficient anti-tumor efficacy of immunotherapy has been shown to depend on activated CD8^+^ T cells which enhance ferroptosis-specific lipid peroxidation in tumor cells [99].

ROS have also been involved in T cell subset differentiation. For example, treatment with pro-oxidants prevented pathogenic Th17 and Th1 cells from producing IL-17 and IFN-γ, respectively [100]. High ROS levels in the environment favored skewing towards a Th2 phenotype [101,102], whereas low levels of ROS have been associated with Th1 and Th17 cell differentiation [101,103], suggesting that targeting T cell redox state may be a potential therapeutic strategy for treating T cell-driven autoimmune diseases. As an example, use of the naturally occurring antioxidant molecule alpha-lipoic acid is being investigated for its immunomodulatory effects for the potential treatment of autoimmune diseases [104].

**Figure 3.**
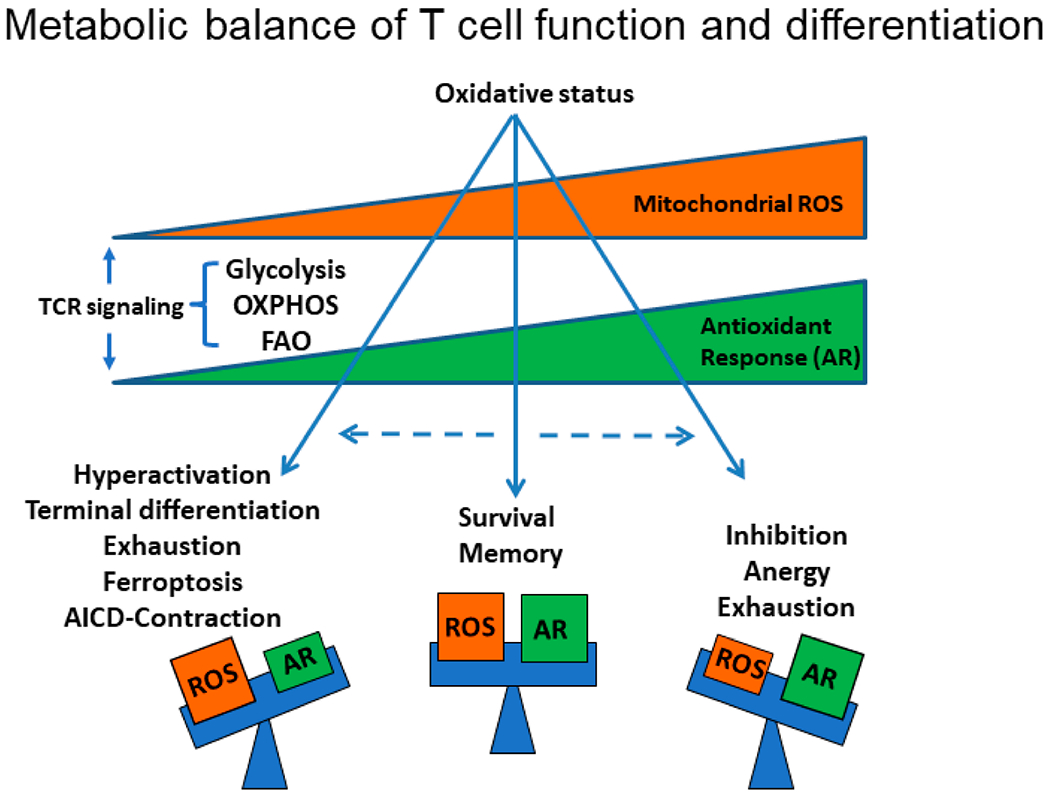
Metabolic balance of T cell function and differentiation. Glycolysis, OXPHOS and FAO are tightly connected with ROS metabolism. TCR signaling simultaneously increases ROS levels and antioxidant responses. Modulating T cell metabolism alters ROS production and redox state with distinct effects on T cell activation, anergy and apoptosis, while antioxidant response (AR) halts T cell responses. ROS levels that exceed the protective capacity of antioxidant response may result in T cell hyperactivation and AICD or ferroptosis associated with cell contraction. Moderate ROS levels in turn are associated with T cell longevity and memory differentiation. Low ROS response levels are associated with T cell hypo-responsiveness and exhaustion.

Although moderate ROS production from mitochondria is required for T cell activation, excessive production has negative effects (Figure 3). It has been reported that T cells displaying high mitochondrial membrane potential produce more ROS and have diminished anti-tumor activity, whilst lower mitochondrial membrane potential is associated with more expression of T memory genes and increased T-cell survival [95]. These observations underline the limitations of using oxidative stress modulators systemically to battle cancer, as it is currently not feasible to precisely control the magnitude of ROS production to optimal, desired levels. Although systemic modulation of oxidative stress alone may not be the right strategy to battle cancer, it might be efficient when synergizing with ICT. PD-1 blockade increased cellular ROS and mitochondrial mass together with proliferation and activation of CD8^+^ T cells in the tumor microenvironment [105]. Pharmacologic increase of ROS either directly by ROS precursors or indirectly by mitochondrial uncouplers synergized with PD-1 blockade to enhance cytotoxic T cell anti-tumor activity. This effect was based on ROS-mediated activation of mitochondrial metabolism through AMPK/mTOR-mediated signaling in tumor-draining lymph nodes, which increased downstream transcription factors such as PGC1α and T-bet [105]. The combination of anti-PD-L1 monoclonal antibody and oltipraz or bezafibrate, two ligands of the PGC1α/Nrf2 and PGC1α/PPAR complexes, respectively, resulted in augmented tumor-suppression activity compared to either treatment alone, explained by the significant increase in both mitochondrial metabolism and glycolysis, driven by PGC1α/PPAR signaling [105]. Other studies showed that high-dose vitamin C in mouse tumor models synergized with anti-PD-1 to enhance cancer immunotherapy, but the exact mechanism of action remains unclear [106,107]. Similarly, adding metformin to PD-1 blockade regimen resulted in enhanced T cell anti-tumor immunity and tumor clearance in murine models by decreasing intra-tumoral hypoxia. This effect might be mediated by inhibition of complex I of the ETC by metformin, which would overall decrease oxygen consumption and OXPHOS [108]. However, metformin is a multifactorial compound, potent activator of AMPK and inhibitor of the glycolytic enzyme hexokinase 2 (HK2) [109,110]. In the context of cancer, these properties of metformin could potentially lead to unwanted effects on T cell differentiation such as AMPK-dependent Treg generation and suppression of glycolytic activity necessary for T cell effector function [39].

### Harnessing Lipid Metabolism to Improve T Cell Function

Lipid metabolism can significantly affect T cell fate and function [111], thus might also be of therapeutic interest in tumor immunotherapy. Effector T cell proliferation and differentiation is supported by fatty acid synthase (FAS) whereas memory CD8^+^ and Treg cells rely on FAO [33,39]. Although manipulating fatty acid metabolism in vivo can affect multiple cell types with unpredictable outcomes, enhancing T cell fatty acid catabolism was shown as a promising therapeutic option in conditions of tumor-mediated T cell exhaustion when T cells were found to highly depend on FAO as the source of energy generation [112]. In fact, promoting FAO by using PPAR-α agonist fenofibrate improved CD8^+^ TIL’s ability and synergized with PD-1 blockade to slow tumor progression and to achieve superior anti-tumor efficacy [112]. This effect could have been linked with enhanced OXPHOS and mitogenic ROS production from mitochondria as supported by similar studies in which treatment with PPAR activator bezafibrate combined with PD-1 blockade but not alone, led to CD8^+^ T cell activation through mitochondrial expansion correlating with decreased tumor growth and increased survival of MC38 tumor-bearing mice [105,113].

High frequencies of intra-tumoral Treg cells represent a major barrier for anti-tumor immunity and tumor immunotherapy [114], but systemic Treg depletion strategies elicit deleterious autoimmune side-effects [115]. A recent study found that genetic ablation of the fatty acid receptor CD36 in Treg cells selectively abrogated the abundance and suppressive activity of intra-tumoral Treg cells without eliciting autoimmunity [116]. The mechanism was dependent on a CD36-PPAR-β signaling axis mediated by unidentified lipids which sustained intra-tumoral Treg survival by maintaining mitochondrial fitness and ETC function, resulting in increased NAD^+^/NADH ratio, which is critical for metabolizing lactate. Targeting CD36 with a monoclonal antibody induced superior anti-tumor immunity, which was further improved when combined with PD-1 blockade. These results suggest that targeting the metabolic adaptation of this newly identified intra-tumoral Treg population might be a promising strategy to improve tumor immunotherapy without disrupting systemic immunity and tissue homeostasis.

Membrane lipids are directly involved in regulation of T-cell signaling and function [117–123]. Previous studies have shown the importance of cholesterol as a key component of membrane lipids, in TCR clustering and immune synapse formation [120–122]. Acetyl-CoA acetyltransferases (ACATs) are cholesterol esterification enzymes that convert free cholesterol to cholesteryl esters for storage. By using melanoma mouse tumor models and either ACAT1 pharmacologic inhibition or ACAT1-knockout mice, a recent study showed increased cholesterol accumulation to CD8^+^ T cell plasma membranes resulting in improved TCR clustering, immunological synapse formation and more potent tumor-killing activity, inhibition of tumor growth, more prolonged survival time, effects that were further improved by PD-1 blockade [124].

### Adoptive Cell Therapy and T Cell Metabolism

Adoptive cell therapy (ACT) is an innovative personalized treatment that involves removal and administration back to the cancer patient of those T cells that have direct anti-tumor activity. The method uses either natural patient T cells with specific anti-tumor activity or patient T cells that have been genetically engineered with specific anti-tumor TCRs or chimeric antigen receptors (CARs) [125]. The functionality of these adoptively transferred cells has also been linked to metabolism [126]. Effective anti-tumor function has been recently shown to be mediated by T cells expressing a hybrid Th1/Th17 phenotype [127]. In melanoma mouse models, hybrid T cells with combined Th1 effector function [128] and Th17 longevity [129–131] had the ability to mediate potent anti-tumor effector function with prolonged survival and persistence. These properties were dependent on the increased levels of NAD^+^ and the elevated activity of NAD^+^-dependent histone deacetylase Sirt1. These observations suggest that pharmacologic intervention to induce generation of such Th1/Th17 hybrid T cells might represent a highly promising approach for improvement of ACT.

Recent studies have identified combinations of costimulatory receptor domains from CD28, 4-1BB, ICOS and/or OX40 to generate CAR T cells with desired metabolic characteristics for long in vivo persistence, resistance to exhaustion and improved effector functions [132,133]. Incorporation of CD28 signal supports T cell activation and is associated with increased glucose influx, higher Glut1 expression, and PI3K/AKT pathway enhancement, leading to augmented glycolytic activity in externally modified effector lymphocytes [26]. 4-1BB costimulatory signal rather induces mitochondrial oxidative metabolism, promoting T memory cell survival, which has been described as an important factor for prolonged in vivo CAR T cell persistence [132,134].

Earlier in vitro studies had shown that engineered T cells to overexpress the antioxidant H_2_O_2_-metabolizing enzyme catalase were resistant to oxidative stress and cell death [135]. Recent in vivo studies from the same group showed that CAR T cells that expressed catalase exerted better anti-tumor activity [136]. Promoting thiol expression can also increase the durability of anti-tumor T cell functions [137]. Consistently, pre-treatment with *N*-acetyl cysteine, which limited AICD and T cell exhaustion, showed significant improvement in the efficacy of adoptive T cell therapy [138,139].

Ex vivo manipulation of lipid metabolism might also be a promising strategy to improve functionality of adoptively transferred T cells. Treatment with cytokines IL-7 or IL-15 would promote lipid metabolism towards FAO to favor memory T cell phenotype [51,52,140,141]. As mentioned above, PPAR agonists that were shown to promote FAO and CD8^+^ TIL function [105,113] or ACAT inhibitors that potentiated T cell function by increasing plasma membrane cholesterol [124] would be expected to synergize with ICT. However, further studies are needed to examine the efficacy and therapeutic potential of these approaches.

Interestingly, overabundance of potassium in the tumor microenvironment can suppress T cell effector function while preserving stemness. High levels of extracellular potassium prevented efficient nutrient uptake, induced autophagy and epigenetic modifications constraining T cell effector programs but favoring in vivo persistence, multipotency, and tumor clearance [142]. It remains to be determined if exposure to increased potassium levels prior to ACT might be a beneficial strategy to improve T cell-based cancer immunotherapy. Manipulating metabolic pathways may provide a strategy to enhance the generation of anti-tumor CD8^+^ T cells with desired memory characteristics and may aid immunotherapy to achieve potent and sustained effects on T cell function (Table 1). Moreover, metabolic conditioning of T cells ex vivo for enhanced mitochondrial capacity and function may improve adoptive cell-based therapies aimed for superior anti-tumor responses (Table 1).

## CONCLUDING REMARKS

Metabolism is undoubtedly tightly linked with T cell fate and function. Signaling molecules, transcription factors and epigenetic regulators are involved in T cell metabolic regulation but are also affected by metabolic alterations. Not only enhanced glycolysis but also balanced mitochondrial function, FAO, OXPHOS and ROS production are critical metabolic determinants of efficient anti-tumor responses. Elucidating the unique and combinatorial role of each metabolic pathway to favor effector, memory or regulatory T cell phenotypes will allow for metabolic interventions at will.

## Figures and Tables

**Table 1.** Potential metabolic interventions for immunotherapy. Potential therapeutic targets and treatments (together with key relevant references) to integrate metabolism in immunotherapy, impact of these potential therapies on the metabolism of T cells and the outcome of each targeted therapy on T cell responses.

Treatment/Targets	Metabolic Impact	Outcome in T cells
PD-1 Blockade [65]	Increased glycolysis	Restored effector function
Compounds promoting ROS production [105]	Activation of T cell transcription factors	Increased effector function
AKT/mTOR inhibitors [37]	Reduced glycolysis	Increased memory generation
PGC1α overexpression, 4-1BB agonist, recombinant Leptin, Bhlhe40 [70,71,73,74]	Enhanced mitochondrial biogenesis and function	Increased memory and enhanced antitumor function
CD36 deletion/inhibition in Tregs[116]	Reduced intratumoral Treg survival	Enhanced antitumor activity

Exposure to increased [K^+^] prior to ACT [142]	Depleted cytoplasmic Ac-CoA, reduced epigenetic modification of effector genes	Maintenance of stemness and long-term persistence
IL-7 and IL-15 prior to ACT [52,140]	Promote Mitochondrial metabolism	Increased memory and in vivo longevity

4-1BBζ CAR T cells [132]	Increased FA oxidation	Increased central memory frequency
CD28ζ CAR T cells [132]	Preferential aerobic glycolysis	Increased effector memory frequency
